# Measuring cancer care coordination: development and validation of a questionnaire for patients

**DOI:** 10.1186/1471-2407-11-298

**Published:** 2011-07-15

**Authors:** Jane M Young, Jennifer Walsh, Phyllis N Butow, Michael J Solomon, Joanne Shaw

**Affiliations:** 1 Cancer Epidemiology and Services Research (CESR), Sydney School of Public Health, Sydney Medical School, University of Sydney, Sydney, NSW, Australia; 2 Surgical Outcomes Research Centre, University of Sydney and Sydney Local Health Network, Sydney, NSW, Australia; 3 Centre for Medical Psychology and Evidence-based Decision Making (CeMPED), School of Psychology, University of Sydney, Sydney, NSW, Australia; 4 Discipline of Surgery, University of Sydney, Sydney, NSW, Australia

**Keywords:** cancer, coordination of cancer care, questionnaire, psychometrics

## Abstract

**Background:**

Improving the coordination of cancer care is a priority area for service improvement. However, quality improvement initiatives are hindered by the lack of accurate and reliable measures of this aspect of cancer care. This study was conducted to develop a questionnaire to measures patients' experience of cancer care coordination and to assess the psychometric properties of this instrument.

**Methods:**

Questionnaire items were developed on the basis of literature review and qualitative research involving focus groups and interviews with cancer patients, carers and clinicians. The draft instrument was completed 686 patients who had been recently treated for a newly diagnosed cancer, including patients from metropolitan, regional and rural areas of New South Wales, Australia. To assess test-retest reliability, 119 patients completed the questionnaire twice. Unreliable items those with limited variability or high levels of missing data were eliminated. Exploratory factor analysis was conducted to define the underlying factor structure of the remaining items and subscales were constructed. Correlations between these and global measures of the experience of care coordination and the quality of care were assessed.

**Results:**

Of 40 items included in the draft questionnaire, 20 were eliminated due to poor test-retest reliability (n = 4), limited response distributions (n = 8), failure to load onto a factor (n = 7) or detrimental effect on the internal consistency of the scale (n = 1). The remaining 20 items loaded onto two factors named 'Communication' and 'Navigation', which explained 91% of the common variance. Internal consistency was with high for the instrument (Cronbach's alpha 0.88) and each subscale (Cronbach's alpha 0.87 and 0.73 respectively). There was no apparent 'floor' or 'ceiling' effect for the total score or the Communication subscale, but evidence of a ceiling effect for the Navigation subscale with 21% of respondents achieving the highest possible score. There were moderate positive associations between the total score and global measures of care coordination (r = 0.57) and quality of care (r = 0.53).

**Conclusions:**

The instrument developed in this study demonstrated consistency and robust psychometric properties. It may provide a useful tool to measure patients' experience of cancer care coordination in future surveys and intervention studies.

## Background

Effective coordination of care between different clinicians, services and health sectors throughout the patient journey is fundamental to the provision of high-quality care [1-3]. In health systems where care is well coordinated, patients will experience effective flow of information between clinicians throughout the course of their illness, with streamlined service provision in response to their physical, emotional and social needs [4]. Not only is good care co-ordination essential to optimize patients' experience, but it has also been shown to reduce future need for supportive care and to improve psychosocial outcomes [5].

People with cancer are particularly at risk of receiving poorly organized and fragmented care due to the complex nature of the disease and its management, which often involves multidisciplinary care from a large team of medical, nursing and allied health practitioners in both hospital and community settings over extended periods of time. As a result, many national strategic cancer plans have identified the improvement of cancer care coordination as a priority for service improvement [1,4,6,7].

Efforts to improve cancer care coordination to date have been hindered by a dearth of accurate and reliable measures by which progress can be monitored. This partly stems from the lack of an agreed theoretical framework or definition of the term 'care coordination' to underpin the development of measures. For example, a recent literature review prepared for the Agency for Healthcare Research and Quality (AHRQ) identified more than 40 different definitions for 'care coordination'. However, the authors identified a number of common elements to inform the following working definition:

*'Care coordination is the deliberate organization of patient care activities between two or more participants (including the patient) involved in a patient's care to facilitate the appropriate delivery of health care services. Organizing care involves the marshaling of personnel and other resources needed to carry out all required patient care activities, and is often managed by the exchange of information among participants responsible for different aspects of care *[8].

This definition provides a starting point to identify the specific aspects of the care experience that should be addressed in any measurement tool.

There are a number of sources of data that could be used to assess aspects of cancer care coordination, including administrative health datasets, audits of individual patient records or measures based on the experience of patients or clinicians. Patients are ideally placed to rate the adequacy of cancer care coordination, however, as they are likely to be the only individual present at every encounter with health services. Furthermore, the move towards more patient-centred care in which services are organized around the needs and preferences of individual patients, emphasizes the primacy of measures based on patients' own experience. Therefore we conducted this study to develop a questionnaire for patients to assess their experience of cancer care coordination in the treatment phase of the cancer journey, to define the underlying factor structure of the questionnaire and to conduct initial validation by assessing construct validity, internal consistency and test-retest reliability.

## Methods

### Item generation and development of a draft questionnaire

A literature review was undertaken to identify relevant issues and terminology as well as items and scales within existing instruments that could be used to measure aspects of cancer care coordination [8-13]. The literature review was used to develop a series of open-ended questions that were used in a qualitative study to explore issues in care coordination specific to oncology. Focus groups and semi-structured interviews with 24 patients and carers and 29 clinicians in metropolitan, regional and rural areas of New South Wales (NSW) were undertaken to investigate stakeholders' views of the most important components of cancer care coordination and to identify potential questionnaire items. Full details of this qualitative study are reported elsewhere [14] but in brief, eight components of care were identified as being crucial for effective cancer care coordination, namely, organisation of patient care, access to and navigation through the healthcare system, the allocation of a "key contact" person, recognition and understanding of medical team roles, effective communication and cooperation amongst the multidisciplinary team and other health service providers, delivery of services in a complementary and timely manner, needs assessment and sufficient and timely information for the patient.

The results of the literature review and this qualitative work were used to identify existing items and to generate new items that addressed these eight components of cancer care coordination as well as the concepts espoused in the AHRQ definition [8]. To generate new items, the study team developed statements that addressed the concept in question and sought input from clinicians and other researchers about clarity and wording.

Forty items that related to concepts considered important by a broad range of stakeholders in the qualitative phase and that addressed the theoretical components of cancer care coordination were selected for inclusion in a draft questionnaire. Items were worded both in the positive and the negative with bolding of words used to highlight differences between similar statements. To investigate the most reliable format for response options, two formats were tested. Eighteen items were phrased as statements to which respondents were asked to indicate their level of agreement, using a five-point Likert scale ('Strongly agree', 'Agree', 'Neutral', 'Disagree', 'Strongly disagree'). The remaining 22 items asked about patients' experiences of care in the previous three months, again using a five-point Likert scale ('Never', 'Rarely', 'Sometimes', 'Frequently', 'Always'). A time frame of three months was chosen on clinical grounds to provide a sufficient time window for patients to have received multidisciplinary cancer care. The items with the 'agreement' format were included in random order, followed by the items using the 'experience' format, again in random order. The response option headings were repeated at intervals down the page to break up the lines of text and tick boxes so as to improve the ease of completing the questionnaire. In addition, the questionnaire included two global assessment questions in which respondents were asked to rate firstly, the coordination of their care and secondly, the overall quality of the care they had received, on a scale from one ('Very poor') to ten ('Excellent'). The draft questionnaire was reviewed by clinicians and researchers to assess comprehensiveness of items (face validity) and clarity of wording.

The draft questionnaire was then tested in two separate samples of patients.

### Sample 1

A purposive sample was recruited from six centres (two in Sydney, four in regional New South Wales (NSW)) to provide patients with a range of cancer types, treatment modalities and geographical location. Eligible patients were in follow up for any cancer that had been treated between three and twelve months previously. This time-frame was considered optimal as patients would have experienced the full range of care-co-ordination through the treatment phase of their illness. Patients were considered ineligible if they had insufficient English skills or were cognitively impaired such that they could not complete the questionnaire or were receiving end of life care.

Patients were asked to read and sign a consent form, complete the questionnaire and return these items to the research team in a reply paid envelope. In addition, patients completed items assessing demographic and clinical information, including age; sex; country of birth; marital, education and occupational status; cancer type, year of diagnosis and treatment modalities. To assess test-retest reliability, on receipt of their completed questionnaire, patients in the first three month period of recruitment were mailed a second, identical copy of the questionnaire to complete two weeks later.

### Sample 2

This sample comprised patients with a newly diagnosed colorectal cancer who were participating in an ongoing randomised trial. Patients treated at 22 public and private hospitals in metropolitan and regional centres in NSW were recruited at the time of initial surgical treatment and asked to complete self-administered questionnaires at baseline, one, three and six months. The data for the present study are from the 3-month assessment which included the draft questionnaire about cancer care coordination. Demographic and clinical information was collected at the time of enrolment into the trial.

### Statistical analysis

Characteristics of participants were summarized. For the subsample of Sample 1 who completed the questionnaire twice, test-retest reliability (repeatability) of individual items was assessed by calculating weighted Kappa statistics with 95% confidence intervals (CIs). Items with kappa values of less than 0.40, representing 'fair' or 'poor' agreement,[15] were eliminated from further analyses.

#### Item reduction

Using the combined dataset (n = 686), frequency distributions for each item were examined. Items with more than 5% missing data and those with limited response distributions (70% or more respondents gave the same response) were eliminated [16].

#### Investigation of factor structure

Study data were then randomly split into two equal sized sub-samples. Exploratory factor analysis using squared multiple correlations as prior communality estimates was conducted in each sub-sample separately to assess the consistency of the factor pattern. The principal factor method was used to extract the factors, followed by a promax rotation [17,18]. The number of meaningful factors was determined on the basis of examination of the scree plot, assessment of the proportion of variance accounted for and interpretability of the factors. Factors that explained at least 5% of the common variance were retained. For interpretation of the rotated factor pattern, an item was said to load onto a particular factor if the factor loading was greater than 0.40 for that factor, and was less than 0.40 for the other factors.

#### Development of subscales

The factors were used to develop subscales within the questionnaire. First, the scoring for items worded in the negative were reversed, so that a higher score indicated better care coordination for all items. Items that loaded onto each factor were summed to create factor-based scales. To assess whether any individual items reduced the internal consistency of the total score or individual subscales, item-total correlations were calculated. These statistics provide a measure of the correlation between an item and the sum of the remaining items in the scale, with low values (less than 0.2) [16] indicating that an item is not measuring the same construct as other items. Cronbach's alpha was calculated with each item removed in turn. Where Cronbach's alpha was substantially improved by removal of the item, this item was eliminated from the scale and Cronbach's alpha for the remaining items was recalculated [16]. Values of Cronbach's alpha between 0.7 and 0.9 were considered optimal [16]. Correlations between variables were assessed to determine whether any were highly correlated (r > 0.70) suggesting redundancy.

The distribution of subscale scores and the total score were assessed with descriptive statistics and the proportions of respondents with the highest ('ceiling') and lowest ('floor') scores were calculated. Spearman's rank correlation coefficient was calculated for the total score and each of the subscales firstly with the global cancer care coordination item and secondly with the global quality of care item. All statistical analyses were undertaken using SAS statistical software [19].

### Sample size

A sample of five times the number of questionnaire items is considered the minimum for factor analysis [20]. As the questionnaire contained 40 items, we needed a minimum of 200 patients in each split sample for this analysis. A minimum sample size of 50 is recommended for assessment of test-retest reliability [15].

### Ethics approval

The study was approved by the Sydney South West Area Health Service Ethics Review Committee (RPAH zone).

## Results

Overall, 686 patients completed the questionnaire, 245 from Sample 1 and 441 from sample 2. Characteristics of patients are summarized in Table 1. The mean age of participants was 66.1 years (sd 13.3 years), with a range of 23-98 years.

**Table 1:** Characteristics of respondents

	n	%
Sex		
Male	365	53.2
Female	321	46.8

Country of birth		
Australia	521	75.9
Other	165	24.0

Marital status		
Never married	73	10.6
Married/living as married	459	66.9
Other	143	20.8

Education		
Primary school only or none	68	9.9
Secondary school	340	49.6
Tertiary degree or diploma	242	35.3

Employment status		
Casual/part time/student	60	8.7
Full time	161	23.5
Retired	405	59.0
Unemployed	47	6.9

Location		
Metropolitan	409	59.6
Regional	277	40.4

Cancer Site		
Colorectal	566	82.5
Gynaecological	52	7.6
Breast	18	2.6
Lung/mesothelioma	9	1.3
Other/multiple sites	32	4.7

Cancer type		
Primary cancer	630	91.8
Recurrent cancer	26	3.8

Treatments received		
Surgery	659	96.0
Chemotherapy	278	40.5
Radiotherapy	84	12.2
Hormone therapy	27	3.9

^Where data are missing, categories do not sum to 100%

### Item reduction

Among 119 patients in Sample 1 who completed the questionnaire twice, values of weighted Kappa for individual items ranged from 0.29 to 0.69. Four items with values less than 0.40 were eliminated from further analyses. Two of these used the 'agreement' response format and two the 'experience' response format. Using the entire dataset (n = 686), eight items demonstrated limited response distributions with 70% or more of the sample giving the same response and so were eliminated. These eight items each used the 'experience' response format.

### Construct validity

The underlying constructs represented by the remaining items were explored in factor analysis using the split sample approach. In the first sub-sample, a solution in which 21 items loaded onto two factors had a simple structure with all items having a high loading on one factor and a low loading on the other. Furthermore, each factor contained at least three items, considered the minimum for a robust subscale [19] and the items on each factor shared some conceptual meaning. When repeated using the second sub-sample, a consistent factor pattern emerged and so results from an analysis using the entire dataset are reported. One item was eliminated subsequently as the internal consistency of the questionnaire improved substantially with its removal. One item was retained despite a factor loading of only 0.37 as this item had loaded more strongly in the separate subsamples and because it was the only one that addressed difficulties with access to general practitioners, an issue that had emerged from the qualitative research as being of great concern, particularly for people living in regional and rural areas. The factors were labelled 'Communication' (13 items) and 'Navigation' (7 items) (Table 2). The revised instrument demonstrated good internal consistency with values of Cronbach's alpha greater than 0.7 for both subscales and the total score (Table 3). Item to item correlations ranged from 0.19 to 0.65 for the Communication subscale and from 0.18 to 0.62 for the Navigation subscale.

**Table 2:** Factor structure and loadings

Item	Factor 1	Factor 2
I knew the warning signs and symptoms I should watch for to monitor my health	**0.43**	0.14

I always knew what tests, treatments and follow up were planned for me	**0.70**	0.03

I knew whether chemotherapy or radiotherapy were suitable for me	**0.71**	0.11

I was fully informed about the advantages and disadvantages of any additional treatments (eg radiotherapy, chemotherapy or hormonal therapy) that were relevant to me	**0.75**	0.11

I always knew the reason why I was having a test or treatment	**0.64**	0.00

I had access to all the additional services (eg stoma therapy, counselling, cancer support groups, nutritional advice) that I needed	**0.60**	0.07

I had sufficient help from staff with dealing with the emotional impact of my cancer	**0.60**	0.13

I had a good understanding of the things I was responsible for to help my treatment plan run smoothly	**0.65**	0.02

I had sufficient help from staff with practical arrangements	**0.67**	0.00

I was fully informed by staff about my financial entitlements (eg Medicare and health fund claims, travel allowances etc)	**0.46**	0.17

The health professionals looking after me always picked up on whether I was feeling anxious or down	**0.58**	0.11

How often were you asked how your visits with other health professionals were going?	**0.46**	0.09

How often were you asked how well you and your family were coping?	**0.50**	0.06

How often were you unsure who you should contact if you had concerns about your health or treatment plan?	0.06	**0.68**

How often were you unsure who to call out of business hours if you had a problem?	0.03	**0.68**

How often were you confused about the roles of the different health professionals involved in your care?	0.03	**0.67**

How often was it difficult to meet the financial costs associated with your health care?	0.10	**0.45**

How often did you feel that health professionals looking after you were not fully informed about your history and progress?	0.02	**0.52**

How often did you have difficulty getting an appointment with your GP?	0.03	**0.37**

How often did you have to wait too long to get the first available appointment for a test or treatment?	0.04	**0.43**

Eigenvalue % variance explained	5.9 74.3%	1.4 17.0%

**Table 3:** Internal consistency, inter-item correlations and correlations with other measures

	Communication	Navigation	Total score
Cronbach's alpha	0.87	0.73	0.88

Correlation with global measure of care coordination	0.50	0.47	0.57

Correlation with global measure of quality of care	0.48	0.41	0.53

Although the Navigation subscale demonstrated a ceiling effect with 21% of the sample achieving the highest possible score, the Communication subscale and total score were both more Normally distributed with no evidence of ceiling or floor effects (Table 4). There were moderate positive correlations between the scales and the global measures of care coordination and quality of care (Table 3). The revised questionnaire is available in Additional File 1.

**Table 4:** Distribution of scores

	Communication	Navigation	Total score
Possible range	13-65	7-35	20-100

Mean (sd)	47.9 (8.1)	30.5 (4.2)	78.3 (10.7)

Median (IQR)	48 (43-53)	31 (28-34)	79 (72-85)

'Floor' - % with lowest possible score	0.4	0	0

'Ceiling' - % with highest possible score	1.1	20.9	0

## Discussion

Despite increasing recognition of inadequate care coordination as a common problem experienced by patients, to date there have been few measures by which improvement or deterioration in this crucial aspect of cancer care could be measured. The aim of this study was to develop a valid and reliable self-administered questionnaire for patients to measure the adequacy of cancer care coordination for those in the treatment phase of the cancer journey. The resulting questionnaire demonstrated robust psychometric properties and consistent subscales, suggesting that this instrument could provide a useful tool to measure cancer care coordination in future patient surveys and intervention studies.

The process of developing a new questionnaire is lengthy, requiring a number of iterative steps to further refine the wording and items to provide an instrument that is both acceptable and easily-understood by the target audience as well as providing a comprehensive, accurate and reliable measure of the phenomenon of interest. Furthermore, there is always a tension between the comprehensiveness of the instrument and the burden that a lengthy instrument will place on respondents. Brief instruments may achieve higher response rates, but may also limit the breadth or depth of information that can be collected. The approach to instrument development in this study was to only include items that had sound psychometric properties. Unreliable items that elicit inconsistent responses from an individual are of no value, as are items that are frequently missed out, perhaps due to lack of clarity in the wording or lack of relevance to a significant number of individuals. Furthermore, items that elicit highly skewed responses, with almost everyone giving a similar response, are of limited value for measurement. On the basis of these considerations, the draft questionnaire was reduced to 20 items that demonstrated good internal consistency and addressed two important components of cancer care coordination, namely the issues of communication and navigation of the health care system.

The Communication factor was the strongest, accounting for nearly 75% of the variance and demonstrating internal consistency in the desired range of 0.7-0.9 [16]. Comprising fewer items, the Navigation subscale necessarily had a lower value of Cronbach's alpha as this is partly dependent on the number of items in the scale [16]. Although the response distributions for the total score and Communication subscale were approximately Normal with no evidence of a ceiling effect, this was not the case for the Navigation subscale. There was a marked ceiling effect for this subscale, suggesting it may have limited usefulness as a stand-alone measure. Further development of this subscale, through inclusion and testing of additional items is warranted.

'Care coordination' and 'continuity of care' are related but distinct concepts [21-23]. While 'care coordination' broadly addresses process issues relevant to streamlined and appropriate navigation of the health care system, 'continuity of care' focuses more on consistency of information and clinical management between providers and over time, and on continuity within relationships [21-23]. As a result, measures of continuity of care have often focused on the issue of whether a patient saw the same doctor at each follow up visit [10,24,25]. Although seeing the same doctor is desirable in certain circumstances, for example within a specific clinic, this aspect of care is less relevant for a broad assessment of the coordination of cancer care which is often multidisciplinary in nature, involving consultations with a number of different health professionals where good communication and exchange of information is paramount. Other existing questionnaires have focused on assessment of patients' experience of hospital discharge, in recognition that the risk of poor care coordination is particularly high at times of transition in care [11,26].

In the non-oncology setting, McGuiness and Sibthorpe took a broad approach to measuring health care coordination, developing an instrument for older patients with chronic, complex medical conditions in the primary care setting [12]. Others have included a single or small number of relevant items pertinent to care coordination within questionnaires to assess perceptions of the quality of care or satisfaction with cancer treatment generally, however this approach limits the depth of information gathered specifically about care coordination [27,28] In contrast, our instrument was designed to provide a more comprehensive assessment of cancer care coordination based on the issues identified by patients and clinicians in our previous qualitative research.

Overall, rates of item completion were high, suggesting that the questions were clear and acceptable to patients. Of note, the highest rates of missing data were for items asking about family and carer issues. The reason for this warrants further investigation as it could be that some patients do not have a carer, or do not identify their family or friends as 'carers', or are unable to answer questions relating to the experience of their carers. Assessment of the experience of cancer care coordination from the perspective of carers warrants further research.

A number of limitations to this study are acknowledged. Although our sampling strategy aimed to include a broad range of patients in Sample 1, there was a preponderance of those with colorectal cancer and people from metropolitan centres. Furthermore, people with limited English skills were excluded from both samples and so the questionnaire may not be applicable to those from culturally and linguistically diverse communities. The methods used in this study were to reduce the number of questionnaire items to those with good psychometric properties and to provide a brief questionnaire. It is possible that the resulting instrument omits important aspects of cancer care coordination and the development of additional items and subscales could improve the content validity of the instrument. Furthermore, the responsiveness of this instrument to change needs to be tested in future studies.

## Conclusion

In conclusion, the questionnaire developed in this study has been shown to be a psychometrically robust patient-report measure of cancer care coordination. Further studies will help establish the usefulness of this measure in future needs assessment surveys and intervention studies.

## Competing interests

The authors declare that they have no competing interests.

## Authors' contributions

JY designed the study, conducted the statistical analysis of the data and drafted the manuscript. JW recruited clinicians and patients, conducted the qualitative studies to generate items, managed the data, contributed to interpretation of findings, critically reviewed the manuscript and approved the final version. PB contributed to study design and development of questionnaire items, interpretation of findings, critically reviewed the manuscript and approved the final version. MS contributed to study design and development of questionnaire items, recruited patients, contributed to interpretation of findings, critically reviewed the manuscript and approved the final version. JS contributed to factor analyses and interpretation of the data, critically reviewed the manuscript and approved the final version. All authors read and approved the final manuscript.

## Pre-publication history

The pre-publication history for this paper can be accessed here:

http://www.biomedcentral.com/1471-2407/11/298/prepub

## Supplementary Material

Additional file 1 **Cancer Care Coordination Questionnaire for Patients**. Copy of the 20-item questionnaire for patients. Click here for file
