# Supplementary material for: Measuring cancer care coordination: development and validation of a questionnaire for patients
Source: BMC Cancer. 2011 Jul 15;11:298. doi: 10.1186/1471-2407-11-298 (PMC3151230; doi:10.1186/1471-2407-11-298)
Supplement: Additional file 1 — Cancer Care Coordination Questionnaire for Patients. Copy of the 20-item questionnaire for patients. [file 1471-2407-11-298-S1.DOC]

**Cancer Care Coordination Questionnaire for Patients**

The following questions ask about your health care experience over the past 3 months. The questions are about the care that you have received from all the health professionals who have been looking after you during your treatment.

These health professionals include your surgeon, GP and anyone else who has been involved in your care such as a care coordinator or cancer nurse, community nurses, a medical oncologist (if you have had chemotherapy) or radiation oncologist (if you have had radiotherapy), a stoma therapist, physiotherapist, psychologist or counsellor.

For each of the following questions, please tick the box for the response that best applies to you.

|  |  | **STRONGLY DISAGREE** | **DISAGREE** | **NEUTRAL** | **AGREE** | **STRONGLY AGREE** |
| --- | --- | --- | --- | --- | --- | --- |
| 1 | I knew the warning signs and symptoms I should watch for to monitor my health | ❑_1_ | ❑_2_ | ❑_3_ | ❑_4_ | ❑_5_ |
| 2 | I always knew what tests, treatments and follow up were planned for me | ❑_1_ | ❑_2_ | ❑_3_ | ❑_4_ | ❑_5_ |
| 3 | I knew whether chemotherapy or radiotherapy were suitable for me | ❑_1_ | ❑_2_ | ❑_3_ | ❑_4_ | ❑_5_ |
| 4 | I always knew the reason why I was having a test or treatment | ❑_1_ | ❑_2_ | ❑_3_ | ❑_4_ | ❑_5_ |
| 5 | I was fully informed about the advantages and disadvantages of any additional treatments (eg radiotherapy, chemotherapy or hormonal therapy) that were relevant to me | ❑_1_ | ❑_2_ | ❑_3_ | ❑_4_ | ❑_5_ |
| 6 | I had access to all the additional services (eg stoma therapy, counselling, cancer support groups, nutritional advice) that I needed | ❑_1_ | ❑_2_ | ❑_3_ | ❑_4_ | ❑_5_ |
| 7 | I had sufficient help from staff with dealing with the emotional impact of my cancer | ❑_1_ | ❑_2_ | ❑_3_ | ❑_4_ | ❑_5_ |
| 8 | I had a good understanding of the things I was responsible for to help my treatment plan run smoothly. | ❑_1_ | ❑_2_ | ❑_3_ | ❑_4_ | ❑_5_ |
| 9 | I had sufficient help from staff with practical arrangements | ❑_1_ | ❑_2_ | ❑_3_ | ❑_4_ | ❑_5_ |
| 10 | I was fully informed by staff about my financial entitlements (eg Medicare and health fund claims, travel allowances etc) | ❑_1_ | ❑_2_ | ❑_3_ | ❑_4_ | ❑_5_ |
| 11 | The health professionals looking after me always picked up on whether I was feeling anxious or down | ❑_1_ | ❑_2_ | ❑_3_ | ❑_4_ | ❑_5_ |

The following questions ask about your experiences over the past 3 months.

|  |  | **NEVER** | **RARELY** | **SOMETIMES** | **FREQUENTLY** | **ALWAYS** |
| --- | --- | --- | --- | --- | --- | --- |
| 12 | How often were you asked how your visits with other health professionals were going? | ❑_1_ | ❑_2_ | ❑_3_ | ❑_4_ | ❑_5_ |
| 13 | How often were you asked how well you and your family were coping? | ❑_1_ | ❑_2_ | ❑_3_ | ❑_4_ | ❑_5_ |
| 14 | How often were you unsure who you should contact if you had concerns about your health or treatment plan? | ❑_1_ | ❑_2_ | ❑_3_ | ❑_4_ | ❑_5_ |
| 15 | How often were you unsure who to call out of business hours if you had a problem? | ❑_1_ | ❑_2_ | ❑_3_ | ❑_4_ | ❑_5_ |
| 16 | How often were you confused about the roles of the different health professionals involved in your care? | ❑_1_ | ❑_2_ | ❑_3_ | ❑_4_ | ❑_5_ |
| 17 | How often was it difficult to meet the financial costs associated with your health care? | ❑_1_ | ❑_2_ | ❑_3_ | ❑_4_ | ❑_5_ |
| 18 | How often did you feel that health professionals looking after you were not fully informed about your history and progress? | ❑_1_ | ❑_2_ | ❑_3_ | ❑_4_ | ❑_5_ |
| 19 | How often did you have difficulty getting an appointment with your GP? | ❑_1_ | ❑_2_ | ❑_3_ | ❑_4_ | ❑_5_ |
| 20 | How often did you have to wait too long to get the first available appointment for a test or treatment? | ❑_1_ | ❑_2_ | ❑_3_ | ❑_4_ | ❑_5_ |

**For the following questions please circle the number between 1 and 10 that best applies to you.**

In general, how would you rate the co-ordination of your care?

1 2 3 4 5 6 7 8 9 10

Very poor Excellent

Overall, how would you rate the care you have received?

1 2 3 4 5 6 7 8 9 10

Very poor Excellent
